# Nanoclusters first: a hierarchical phase transformation in a novel Mg alloy

**DOI:** 10.1038/srep14186

**Published:** 2015-09-21

**Authors:** Hiroshi Okuda, Michiaki Yamasaki, Yoshihito Kawamura, Masao Tabuchi, Hajime Kimizuka

**Affiliations:** 1 Department of Materials Science and Engineering, Kyoto University, Sakyo-ku Kyoto 606-8501 Japan; 2 Magnesium Research Center, Kumamoto University, Kurokami Kumamoto 860-8555 Japan; 3 Synchrotron Radiation Center, Nagoya University, Chikusa, Nagoya 464-8601 Japan; 4 Department of Mechanical Science and Bioengineering, Osaka University, Toyonaka 560-8531 Japan

## Abstract

The Mg-Y-Zn ternary alloy system contains a series of novel structures known as long-period stacking ordered (LPSO) structures. The formation process and its key concept from a viewpoint of phase transition are not yet clear. The current study reveals that the phase transformation process is not a traditional spinodal decomposition or structural transformation but, rather a novel hierarchical phase transformation. In this transformation, clustering occurs first, and the spatial rearrangement of the clusters induce a secondary phase transformation that eventually lead to two-dimensional ordering of the clusters. The formation process was examined using *in situ* synchrotron radiation small-angle X-ray scattering (SAXS). Rapid quenching from liquid alloy into thin ribbons yielded strongly supersaturated amorphous samples. The samples were heated at a constant rate of 10 K/min. and the scattering patterns were acquired. The SAXS analysis indicated that small clusters grew to sizes of 0.2 nm after they crystallized. The clusters distributed randomly in space grew and eventually transformed into a microstructure with two well-defined cluster-cluster distances, one for the segregation periodicity of LPSO and the other for the in-plane ordering in segregated layer. This transformation into the LPSO structure concomitantly introduces the periodical stacking fault required for the 18R structures.

Metallic alloys undergo various types of phase transformations. These processes, including spinodal decomposition^1^, clustering^2^, order-disorder transition accompanying spinodal decomposition^3^, and displacive transformation^4^, have been the subjects of intense interest from a statistical thermodynamics viewpoint^5^,^6^ and from that of strategies for developing innovative materials. An understanding of these mechanisms can be extended to a much wider range of self-organized nanostructures, such as semiconducting nanodot heterostructures^7^, block copolymers^8^ and complex fluids^9^. We demonstrate that novel light weight Mg alloys with industrial interest^10^, Mg-Y-Zn ternary alloys, exhibit a novel spontaneously hierarchical phase transition, first clustering and second crystallization of clusters, i.e., ordering of the spatial arrangements of clusters, ultimately leading to order-disorder (OD) structures^11^,^12^.

An emerging group of Mg alloys, with a series of novel structures called synchronized long-period stacking ordered (LPSO) structures^13^,^14^, have attracted much attention for two reasons. One is interest in how and why such strange structure is stabilized, i.e., from phase transformation viewpoints. The other reason is interest in their mechanical properties, i.e., higher strength for practical applications ranging from mobile computers to aircrafts, and basic questions about the kink deformation mechanism^15^,^16^.

The LPSO phase was first observed in MgZnY alloys as a periodic concentration modulation of Zn and Y along the c axis^13^,^14^ in an hcp Mg matrix, and then a more refined structure was determined by electron microscopic works focusing on determining equilibrium (stable) structures of LPSO phases^17^,^18^. A reported structure^17^ is schematically illustrated in Fig. 1. The segregation layers consist of a two-dimensionally ordered arrangement of L1_2_-type Y_8_Zn_6_ or Gd_8_Al_6_ molecular-like clusters with 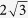 × 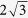 superstructures^17^, whose stacking in the perpendicular direction is described by crystallographic operations^12^. The structure appears similar to that of intermetallic compounds with well-defined atomic positions and occupancies. However, upon examining the temporal structural change using synchrotron radiation small-angle scattering (SR-SAXS), we observed that the distance between the ordered clusters changed continuously with time^19^,^20^ within the segregation plane. The in-plane structure shown in Fig. 1 appears to undergo a two-dimensional order-disorder transition of clusters^21^. Such superstructures by clusters in the segregation layer might be examined in terms of two-dimensional phase transformations of particles^22^ similar to colloidal crystals. The confinement of the L1_2_-ordered clusters occurs in two-dimension, as if the clusters, a partial structure of a complex order structure, behave as an independent structure unit. It should be noted that the system is three-dimensional, and the formation and growth of such clusters are driven by the same phase transformation process. Knowing this, we need to return to the question of how we can understand the stability and formation mechanism of the LPSO structures in three dimension in relation to the two-dimensional order-disorder behaviour.

In many metallic alloys, a phase transition is observed upon quenching materials from a high temperature (higher symmetry) phase into a supersaturated state. For example, by quenching Al alloys, clusters of a minority phase called Guinier-Preston (GP) zones^23^,^24^ develop, whose characteristic lengths, such as their average sizes, grow with a temporal power law^25^,^26^. Comparison of the growth power law with the prediction from cluster dynamics theories or simulations^27^ provides an image of the transformation, such as the centre of gravity of the nanoclusters moving (diffusion of clusters), and their coagulation during the early stage of precipitation. When the volume fraction of a minority phase is large, spinodal decomposition^1^ or spinodal ordering^3^ is expected. For the present Mg_85_Y_9_Zn_6_ alloys, thermodynamical analysis^28^ suggested possibility of spinodal decomposition^1^ as the major driving force of LPSO formation. However, the periodic stacking faults in the LPSO structures, as shown in Fig. 1 starts another discussion from a structural phase transformations viewpoint, i.e., stacking-fault driven transformation^29^. However, neither of the mechanisms appears to be compatible with the two-dimensional ordering kinetics of the present alloys described above.

## Results and Discussions

To examine the initial stage of the formation process, small-angle X-ray scattering (SAXS) patterns were measured *in situ* while heating rapidly quenched Mg_85_Y_9_Zn_6_ ribbons. The sample ribbons were initially glassy and uniform at the macroscopic length scale. A single-phase 18R LPSO microstructure is reported for the composition after long-time annealing.

**Figure 2:**
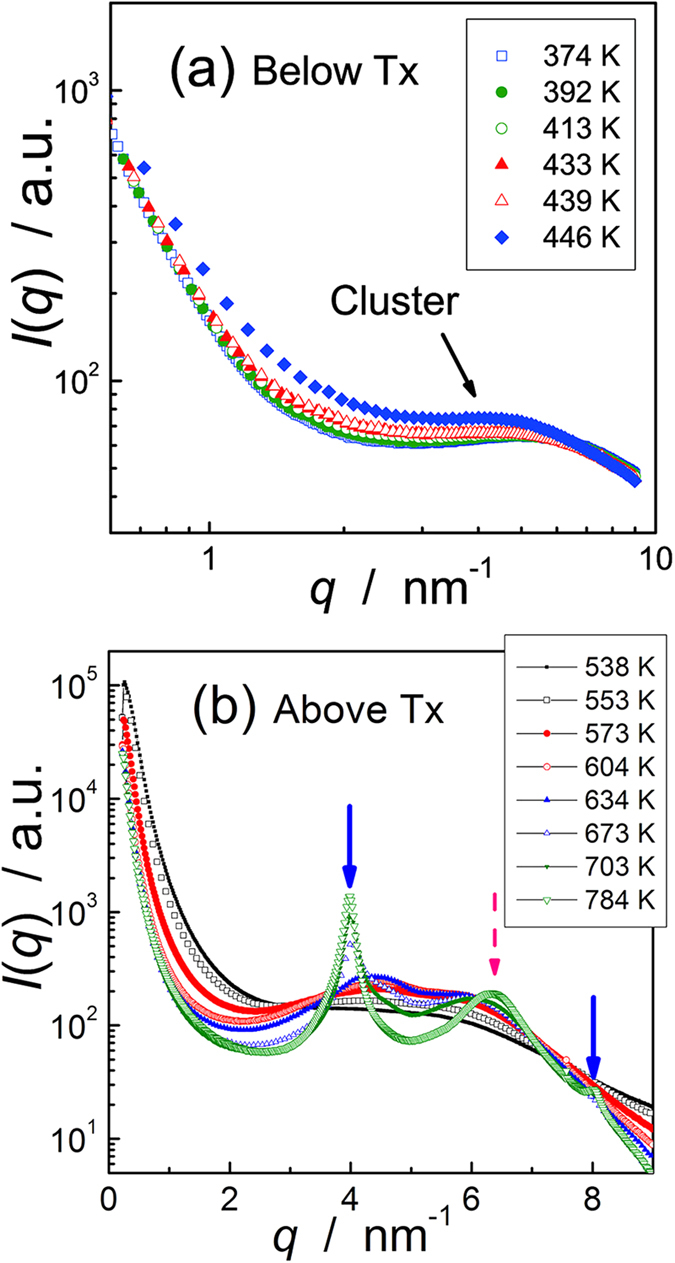
*In-situ* small-angle scattering during heating (**a**) below and (**b**) above the crystallization temperature, T_x_. The shoulder denoted as cluster in (**a**) represents scattering by small clusters distributed isotropically in space. At higher temperature (**b**), a diffuse peak in (**a**) decomposed into two peaks, one for 18R concentration modulation and the other for in-plane ordering. The clear peaks at approximately 4 nm^−1^ and 6 nm^−1^ are the fingerprint of the 18R LPSO structure.

**Figure 3:**
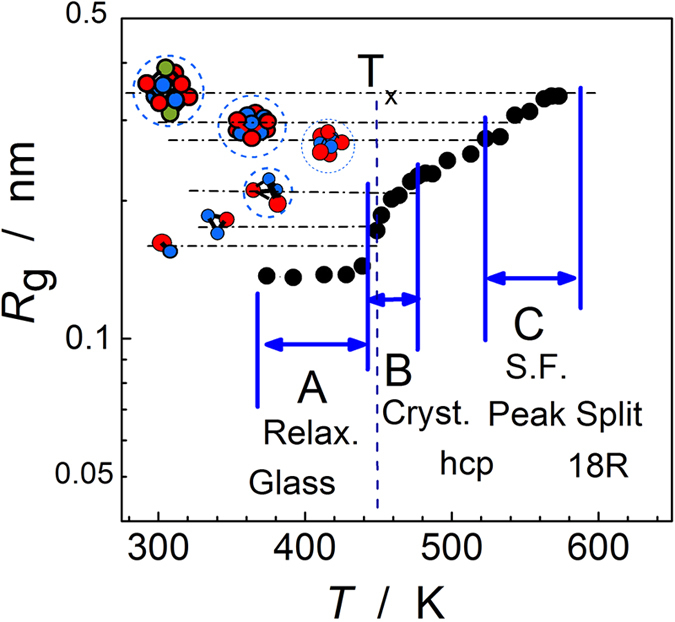
Cluster size determined from *in situ* SAXS. The size of the clusters remained at 0.14 nm in the glass structure and then rapidly increased to 0.2 nm upon crystallization (T_x_). The growth slowed down when the size reached that of the L1_2_ cluster in stable 18R structure.

Figure 2 shows the change in the SAXS profile during heating. The round shoulders of the scattering intensities in the figure indicate that many small clusters stably exist even at low temperatures, and grow as the temperature increases. Another important point is that the scattering pattern changes the shape from a single diffuse peak to a double sharp peak profile at higher temperatures. With a cluster model, a scattering profile is approximated by the cluster component (form factor, *F*(*q*)) and the spatial arrangements of the clusters (structure factor, *S*(*q*) ), as:


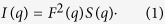


where *q* is the magnitude of scattering vector. The average radius obtained from the form factor is shown in Fig. 3. It is approximately 0.14 nm before crystallization, and a rapid increase to a radius of approximately 0.2 nm was observed when crystallization occurred. The cluster size increased with temperatures, and the growth slowed down at the radius of 0.34 nm at 570 K. The gyration radius of the L1_2_ cluster obtained from a first-principles calculation of the MgYZn LPSO structure is 0.34 ± 0.01 nm, which implies that the early stage of the phase transition should be described by a cluster image, not by classical spinodal decomposition.

**Figure 4:**
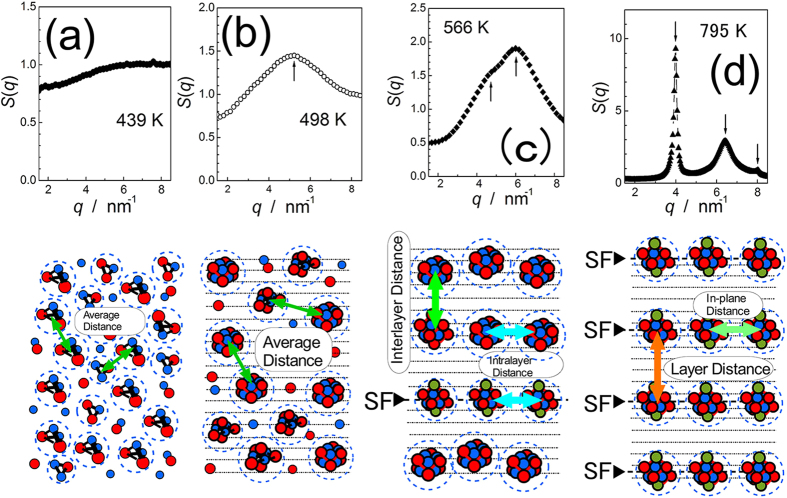
Structure factors and corresponding schematic illustrations of the microstructure during heating. After crystallization, the structure factor results in a well-defined single diffuse peak, suggesting isotropic distribution of the clusters. The average distance between the clusters splits into two groups of distances, one leading to the interlayer distance and the other to the intralayer (in-plane) distance.

Figure 4 shows the structure factors for several temperatures and corresponding schematic illustrations of the nanostructures in the sample. At lower temperatures, the structure factor is characterized by a single diffuse peak, suggesting that the clusters are distributed isotropically in the amorphous or crystal matrix. At higher temperatures above 553 K, the peak of the structure factor begins to separate into two peak components, eventually split into well-defined two sharp peaks corresponding to reported compositional modulation for 18R at 4 nm^−1^ and an in-plane 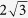 × 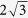 peak at 6.4 nm^−1^ 30. This transition indicates that the formation of LPSO is explained in terms of spatial rearrangements of isotropically distributed ordered clusters into two classes of orders, one corresponds to long-range compositional modulation in the direction of the c-axis to form 18R, and the other corresponds to the two-dimensional ordering leading to a 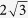 × 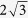
superstructure. We may emphasize that L1_2_ clusters in stable (equilibrium) LPSO structures need to have stacking faults, on which the clusters are pinned. For smaller clusters, mobile clusters without stacking faults are reasonable. The model clusters depicted in Fig. 3 are on an hcp lattice except L1_2_, and the gyration radii indicated by the horizontal lines are determined from the optimized structures using first-principles calculations^21^. Such clusters on an hcp lattice do not need to drag stacking faults upon migration; therefore, the clusters can move and grow freely in the hcp lattice similar to clusters in Al-Zn. The difference between the two alloys is that the clusters in LPSO stopped growing at the size of Y_8_Zn_6_, and formed cluster crystals like colloid crystals with two characteristic distances, an interlayer and intralayer distances. After such separation started above 530 K, where the clusters align in a plane, cooperative shear transformed the hcp-based clusters with DO_19_ into fcc-based clusters with L1_2_. Using first-principles calculations, the energy gain associated with the transformation from Y_6_Zn_6 _+ Y_2_ clusters on an hcp lattice into a Y_8_Zn_6_ L1_2_ cluster was estimated to be at least 1.86 eV per L1_2_ cluster. Assuming a 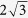 × 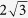 and even coarser superstructure, this energy gain sufficiently overcomes the energy loss caused by stacking-fault formation in the surrounding Mg matrix. Then the clusters are pinned by the stacking fault and two-dimensional self-organization^20^,^21^ begins. An interesting point here is that the clusters of Y and Zn are stable enough to exist even in amorphous state, but never grow larger than the size of approximately one unit cell, resulting in a stable size of molecular-like Y_8_Zn_6_ clusters in the crystalline state. Sharp diffraction peaks from concentration modulation for the periodicity of 18R up to the second order peak at 8 nm^−1^ and in-plane diffraction peak agreed with those for cast samples annealed for long time, whose structure has been well examined by TEM^10^,^14^,^18^ are the signature of formation of 18R structure.

We may refer to two systems that exhibit similar nanostructures: perforated structures in block copolymer systems^31^ and confined colloidal crystal systems^22^. A perforated nanostructure in a block copolymer forms two-dimensional ordered layers self-organized in three-dimensional materials, however, it is different from the present case in that further two-dimensional ordering transition is not observed. Confined colloidal crystals do exhibit two-dimensional phase transition, however, the origin of confinement is external. In contrast, the clusters in the present alloy grows and voluntarily form two-dimensional cluster layers, and then the two-dimensional order of the cluster proceeds, finally leading to interlayer (OD) ordering. An interesting point here is that these hierarchical transitions are driven by the single free energy curve of the LPSO alloy, with a different magnitude of interaction depending on the hierarchy. In short, a strong phase separation tendency^29^, repulsive, but much weaker interaction between the clusters^22^, and even weaker energy gain with OD structures are the key to understand the present case. Further systematic investigation of the related alloy will provide us with a perspective on alloy design and help determine how the magnitude of interaction be implemented to design building blocks in artificial nanostructures such as functional colloidal crystals.

In summary, we demonstrated that the key to understand the formation of LPSO in the present alloy is neither typical spinodal decomposition^1^,^3^ nor structural transformation^4^. The kinetics indicated a hierarchical phase transformation sequence of clustering first, followed by cluster motion for long-period structures with the introduction of stacking faults. Finally two-dimensional nanoscopic ordering in segregation layers was observed.

## Methods

Small-angle X-ray scattering (SAXS) measurements were applied to examine the nanoscopic heterogeneity in the sample. Mg_85_Y_9_Zn_6_ alloy, the composition for single phase 18R LPSO, was rapidly quenched (RQ) from a liquid into amorphous ribbons. The ribbons were heated at a speed of 10K/min. from room temperature to melting in a furnace with carbon insulation and evacuated using a turbo molecular pump. SAXS profiles were acquired every 15 to 20 seconds *in situ* during heating. Calorimetric measurements were made separately to detect the transformation temperatures. XAFS measurements were preformed for as-quenched and annealed ribbons at the K absorption edge of Zn and Y, and were compared with the results for cast ingots annealed at 673 K for a month. The ribbons were isothermally annealed for 5 minutes in sealed Pyrex® tube at the temperatures between 423 K and 582 K. The energetics and equilibrium structure of Mg-Y-Zn systems containing a solute cluster were obtained from first-principles density functional theory (DFT) calculations using the VASP code^32^.

## Additional Information

**How to cite this article**: Okuda, H. *et al.* Nanoclusters first: a hierarchical phase transformation in a novel Mg alloy. *Sci. Rep.* **5**, 14186; doi: 10.1038/srep14186 (2015).

## Figures and Tables

**Figure 1:**
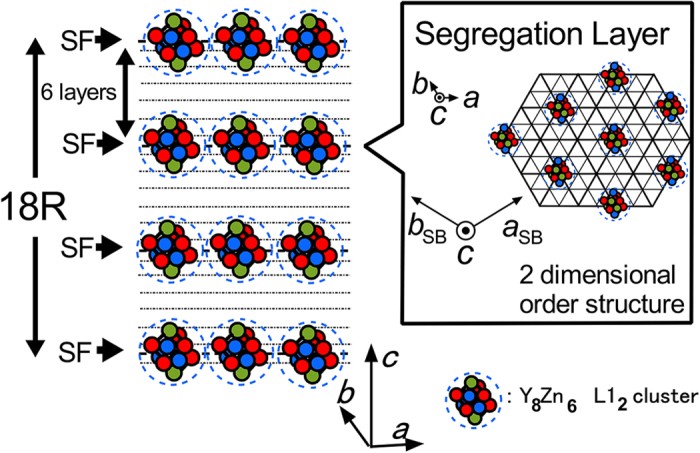
Schematic illustration of a fully ordered 18R structure reported by Yokobayashi *et al.* for MgGdAl alloys. L1_2_ clusters form a 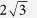
× 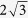 order structure at the stacking faults (SFs). For the MgYZn LPSO structure, Gd is replaced by Y and Al is replaced by Zn. Y atoms occupy 8 corner positions and Zn atoms occupy the face-centre positions for a Y_8_Zn_6_ cluster.
